# Facile synthesis of spongy NiCo_2_O_4_ powders for lithium-ion storage

**DOI:** 10.1038/s41598-023-37315-6

**Published:** 2023-06-23

**Authors:** H. Mahboubi, S. M. Masoudpanah, S. Alamolhoda, M. Hasheminiasari

**Affiliations:** School of Metallurgy & Materials Engineering, Iran University of Science and Technology (IUST), Tehran, Iran

**Keywords:** Batteries, Materials science, Chemical engineering

## Abstract

Spongy NiCo_2_O_4_ powders were prepared by solution combustion synthesis (SCS) method for lithium ions storage. The effects of combustion parameters including fuel type (l-lysine, glycine, and urea) and fuel amount on the lithium storage performance of NiCo_2_O_4_ powders were analyzed by various characterization techniques. Single-phase NiCo_2_O_4_ powders with extremely porous microstructure showed a strong drop of initial specific capacity up to 350 mAhg^−1^ which was recovered up to 666 mAhg^−1^ following 100 charge/discharge cycles. However, the NiCo_2_O_4_ powders prepared by the urea and l-lysine fuels with the compacted microstructure showed the capacity loss without any recovery. The spongy NiCo_2_O_4_ powders showed an acceptable capability rate performance (404 mAhg^−1^ @ 400 mAg^−1^).

## Introduction

High-energy density lithium-ion batteries (LIBs) are one of the most important choices for electric vehicles (EVs) which are in urgent demand on account of fossil fuel depletion and air pollution^1,2^. Despite all the advantages of traditional graphite anode, it suffers from low theoretical capacity (372 mAhg^−1^) and poor safety issues caused by the dendrite formation^3,4^. Among various anode materials, transition metal oxides (TMOs) such as NiCo_2_O_4_, MnO_2_, Fe_3_O_4_, CoFe_2_O_4_, etc. are attracted a great attention as next-generation negative candidates due to the suitable lithiation potential and higher theoretical capacity^5^. The TMOs store electrochemical energy by redox reactions as conversion mechanism due to the absence of vacancy in crystal structure for hosting the Li^+^ ions. The large volume changes of expansion and contraction during the redox reactions lead to the pulverizing and falling off active materials from current collectors, continuously fading capacity via hurting electrical contacts^6^. Previous reports showed that the large volume change limits the application of conversion-type TMOs^7,8^.

Among various TMOs, nickel cobalt oxide (NiCo_2_O_4_) with spinel cubic structure has been extensively considered as a promising anode material for LIBs owing to its high theoretical capacity (891 mAhg^−1^), higher electrical conductivity electrochemical activity than the corresponding monometallic oxides, NiO and Co_3_O_4_^9–11^. Several strategies including compositing with carbon fibers, carbon nanotubes, and graphene/graphene oxide and tuning the morphology have been proposed for improving the cycling performance of NiCo_2_O_4_ anode^12–15^.

One main goal of this work is to develop a facile, simple, and efficient synthesis method for preparation the porous NiCo_2_O_4_ material. It is expected that the porous materials show the better cycling performance by tolerating the volume changes during charging/discharging cycles, in addition to facilitating the ions diffusion and easily accessing the electrolyte via pores^16–19^.

In this work, the spongy NiCo_2_O_4_ powders were prepared by solution combustion synthesis (SCS) method which is based on an exothermic reaction between organic fuels and metal nitrates as oxidants^12^. A large volume of gases is liberated by burning the organic fuels, leading to the high specific surface areas and large pores^20,21^. Furthermore, the effects of the amounts and types of organic fuels including glycine, L-lysine, and urea on the structural, microstructural, and electrochemical properties of NiCo_2_O_4_ powders were explored to clarify the capacity fading mechanism.

## Experimental procedures

### Synthesis method

Ni(NO_3_)_2_·6H_2_O and Co(No_3_)_2_·6H_2_O as oxidants and glycine (C_2_H_5_NO_2_), l-lysine (C_6_H_14_N_2_O_2_), and urea (CO(NH_2_)_2_) as organic fuels were provided by Merck Co.

Firstly, 1 mmol Ni(NO_3_)_2_·6H_2_O and 2 mmol Co(No_3_)_2_·6H_2_O and 30 mmol organic fuels were dissolved in 30 mL distilled water. For examining the effects of fuel amounts, the different amounts (molar ratio) of the intended fuel [1.5 (0.5), 3 (1), and 6 (2) mmol] were dissolved in the distilled water. The precursor solutions were homogenized by magnetic stirring. After drying at 90 °C, the gel was combusted on a hot plate by further heating up to 250 °C. The combustion products were then calcined at 450 °C for 1 h with a heating rate of 10 °C min^−1^ in air atmosphere. For easy presentation, the “G1”, “U1”, and “L1” symbols refer to the NiCo_2_O_4_ powders obtained by glycine, urea, and l-lysine fuels at a molar ratio of 1, respectively.

### Materials characterization

The structural properties were analyzed by an X-ray diffractometer (DRON-8, Bourevestnik, Russia) equipped with CuKα radiation (λ = 1.540 Å). The particle size and shape were examined using a scanning electron microscopy (SEM) (Vega II TESCAN, Czech Republic) at 15 kV. For measuring the N_2_ adsorption–desorption isotherms using a BELSORP-mini II, the powders were degassed at 250 °C for 5 h.

### Electrochemical characterization

The working electrode was prepared by pasting a slurry of NiCo_2_O_4_ as active material, polyvinylidene fluoride as binder, and acetylene black as conductive agent (70:15:15 in mass ratio) in N-methyl-2-pyrrolidone on Cu foil. The as-coated foil was then dried at 90 °C in an oven. The mass loading of electroactive material on the Al foil was 1.5–2 mg cm^−2^. The electrochemical performance was characterized using CR2016 coin cells assembled in Ar-filled glovebox, with metallic lithium as counter electrode, 1 M LiFP_6_ solution (EC:DMC) as electrolyte. The galvanostatic discharge/charge tests were performed in 0.01–3 V vs. Li/Li^+^ on a BTS-5 mV 10 mA battery tester (Neware, China) at different current densities. A Radstat10 system (Kianshar Danesh, Iran) was used to record the electrochemical impedance spectra (EIS) in the frequency range of 0.005–50,000 Hz and cyclic voltammetry in the potential range of 0.01–3.0 V at 0.1 mV s^−1^. To observe the microstructure of cycled cells, the active materials were extracted from the coated foil by rinsing it with diethylene carbonate (DEC) in the glovebox.

## Results and discussion

**Figure 1:**
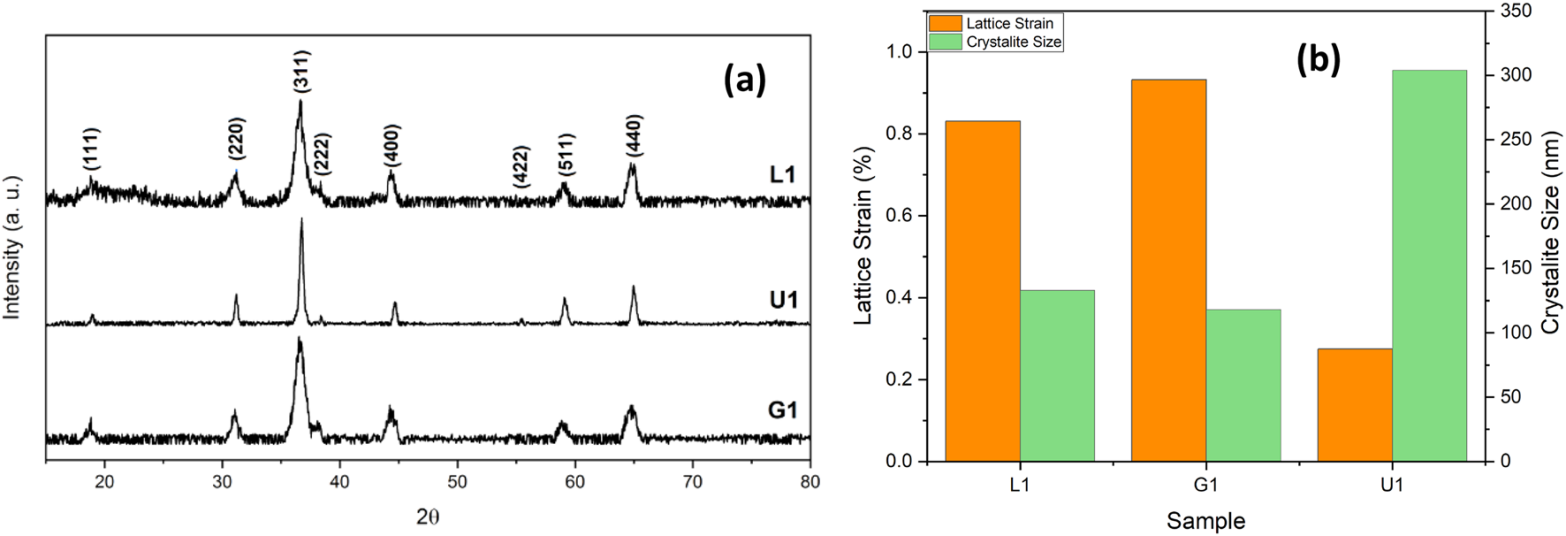
(**a**) XRD patterns, (**b**) lattice strain and crystallite size of the as-calcined powders as a function of fuel type.

Figure 1a shows the XRD patterns of the NiCo_2_O_4_ powders obtained by urea, glycine, and l-lysine fuels. All diffraction peaks are indexed to the cubic spinel structure of NiCo_2_O_4_ phase with a space group of Fd-3m (PDF2#00-073-1702). The absence of any peaks shows the high phase purity of the synthesized NiCo_2_O_4_ powders. The physicochemical properties of NiCo_2_O_4_ are strongly dependent on the released heat during the burning of organic fuel which is related to the amount and type of organic fuel. The glycine, urea, and l-lysine molecules with the amino (NH_2_) group can release the higher thermal energy, leading to the direct formation of final product. However, the combusted products usually are calcined at higher temperatures to enhance the crystallinity by annihilating the various crystal defects. To evaluate the crystallinity, the dependence of crystallite size and lattice strain on fuel type is presented in Fig. 1b. The U1 powder shows the largest crystallite size of 28 nm, while the G1 and L1 powders have a similar crystallite size of about 11 nm, due to the higher combustion adiabatic temperature by the urea fuel. The released heat and liberated gases determine the combustion adiabatic temperature. Therefore, the lower combustion temperatures by the glycine and l-lysine fuels can be attributed to the higher liberated gaseous products than that of urea fuel during combustion reaction.

**Figure 2:**
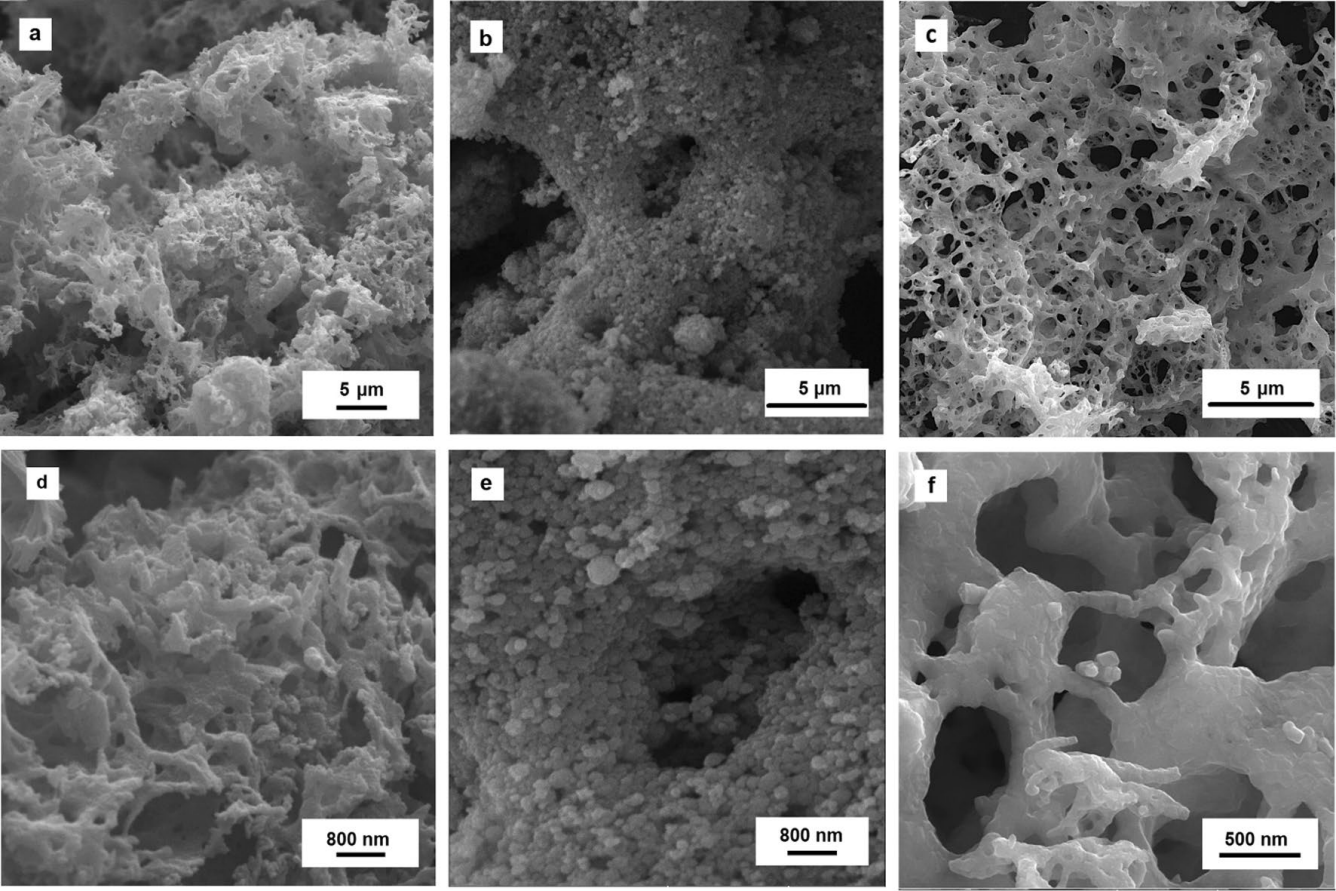
SEM of the as-calcined (**a**, **d**) L1, (**b**, **e**) U1, (**c**, **f**) G1 powders.

Figure 2 compares the SEM images of the G1, U1, and L1 samples. There is a spongy microstructure for the L1 sample (Fig. 2a,d), while the U1 sample (Fig. 2b,e) shows an agglomerated microstructure of spherical particles (~ 245 nm). The G1 powders are composed of an extremely porous microstructure which the NiCo_2_O_4_ nanoparticles (79 nm) are aggregated on the pores’ wall (Fig. 2c,f). The microstructural features such as porosity, particle size, and specific surface area are mainly dependent on the type and amount of the fuel, determining the combustion rate and combustion temperature. A significant amount of large pores in the G1 powders can be attributed to the fast liberation of gaseous products during the combustion reaction. However, some urea molecules can be hydrolyzed to CO_2_ and NH_3_, leading to the initial precipitation of hydroxides during gelation process. Therefore, the lower amounts of released gaseous result in the bulky microstructure for the U1 powders by using the urea fuel^22^.

**Figure 3:**
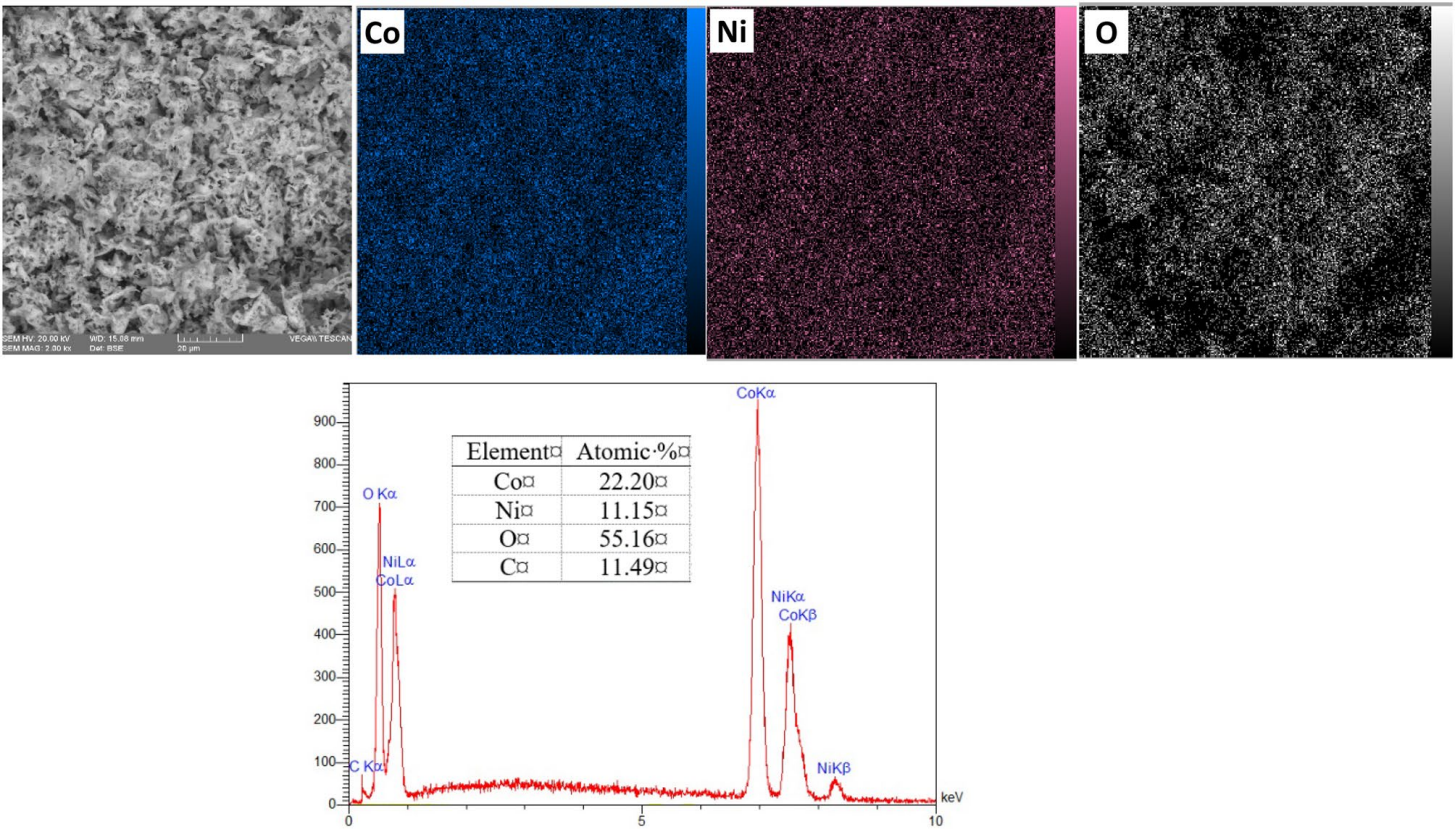
2D distribution of Ni, Co, and O elements and EDS spectrum in the as-calcined G0.5 powders.

The 2D distribution of Ni, Co, O, and C elements and EDS spectrum of as-calcined G0.5 powder (Fig. 3) confirm the uniform distribution of Ni and Co elements. Moreover, the atomic ratio of Co/Ni is approximately 1.96 which is in agreement with the theoretical value of 2.

**Figure 4:**
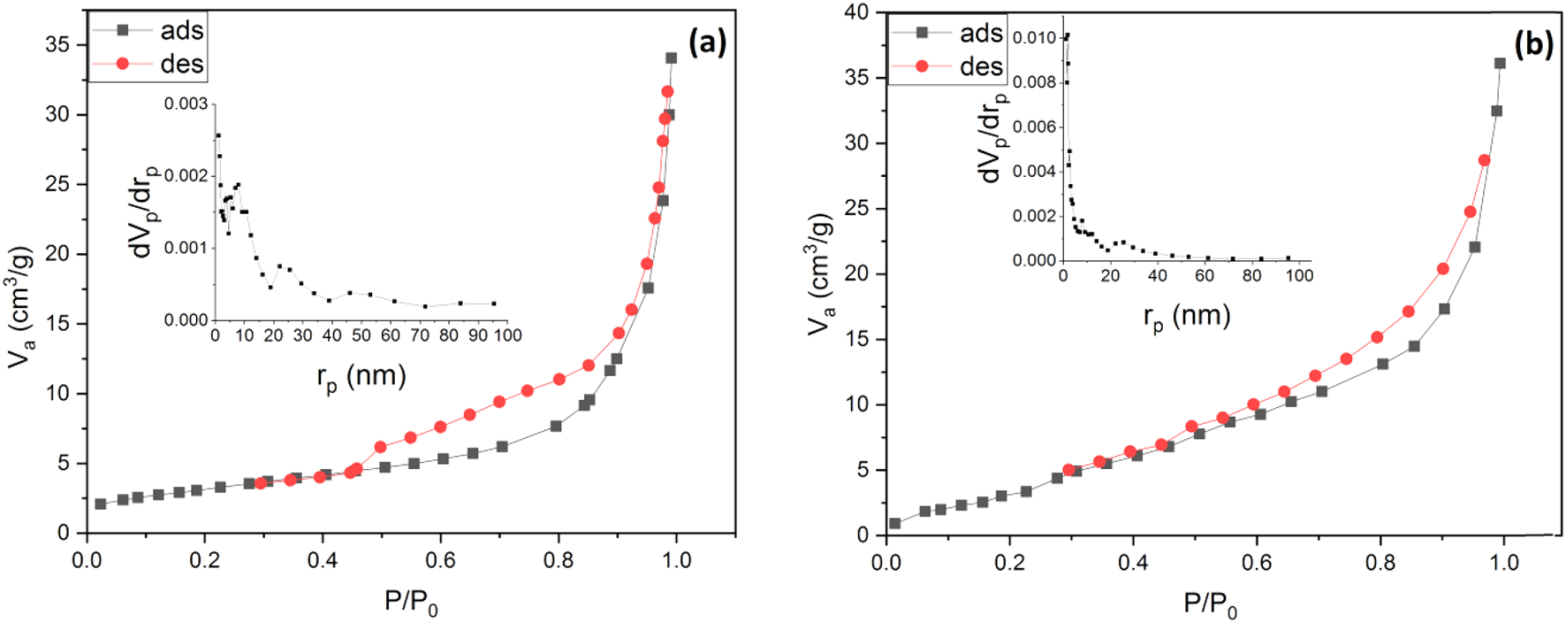
N_2_ adsorption–desorption isotherms and pore size plot of (**a**) U1 and (**b**) G1 powders.

N_2_ adsorption/desorption isotherms and pore size distribution plots of the U1 and G1 powders are compared in Fig. 4a and b. The isotherms are IV type with an H3 hysteresis loop, relating to the brittle agglomerations of particles in a foamy microstructure^23^. The BET specific surface areas of the U1 and G1 powders are 11 and 20 m^2^ g^−1^, respectively. Moreover, the BJH plots show that the pores of both samples are mainly smaller than 40 nm. The higher combustion temperature leads to a lower specific surface area owing to the particles’ growth and sintering. On the other hand, the higher amounts of exhausted gases, the more and the larger pores. Therefore, the higher specific surface areas of the G1 sample is related to the higher amounts of released gaseous products during the combustion reaction^24^.

**Figure 5:**
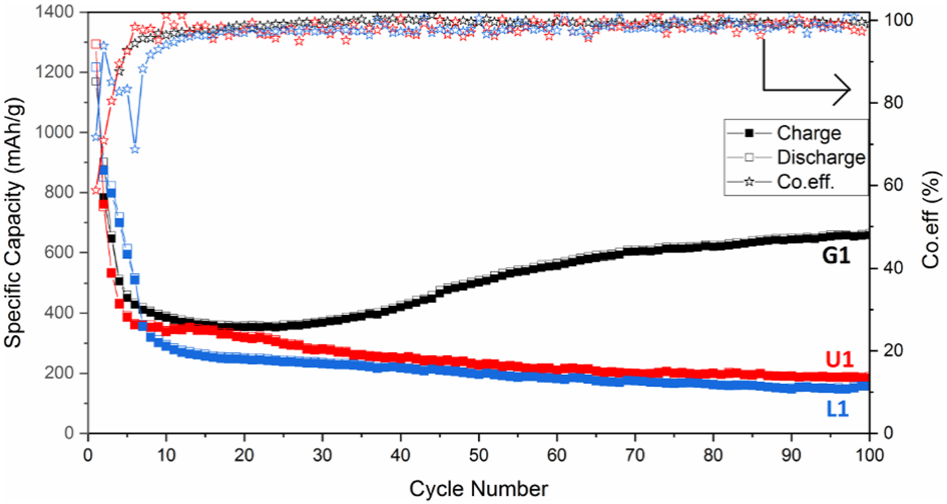
Cycling performance and columbic efficiency of the L1, U1, and G1 powders.

Figure 5 illustrates the cycling performance of the various powders at a current density of 50 mAg^−1^ in the voltage range of 0.01–3.0 V. Although the G1 sample experiences a capacity fading in the initial cycles, it shows a remarkable improvement during the subsequent cycles. After 100 cycles at 50 mAg^−1^, the discharge capacity recovered to 664 mAhg^-1^. However, the cycling performance of the U1 and L1 samples are similar in where the capacity declined and then stabilized at about 200 mAhg^−1^. Due to the superiority of the cycling performance of the G1 sample, the glycine fuel was selected for further studies. Irrespective of fuel type, the columbic efficiency is 100%, indicating the high reversibility of charge/discharge processes.

**Figure 6:**
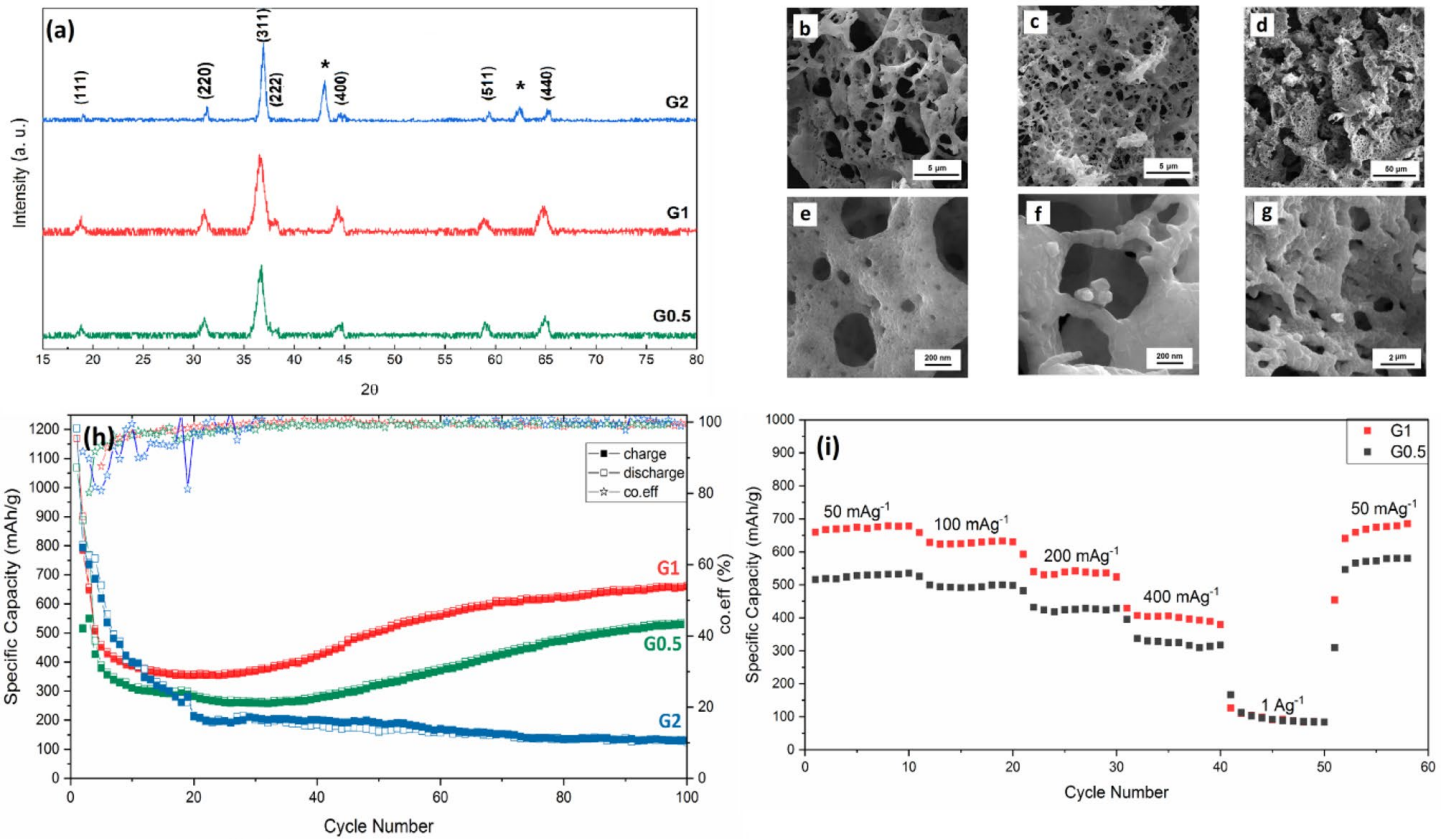
(**a**) XRD patterns and SEM images of (**b**, **e**) G0.5, (**c**, **f**) G1, and (**d**, **g**) G2 powders and (**h**) cycling performance and (**i**) capability rate.

The NiCo_2_O_4_ powders are also prepared at various molar ratios of glycine fuel to total metal precursor which their XRD patterns are compared in Fig. 6a. All diffraction peaks of the G1 and G0.5 samples are related to the cubic spinel structure of NiCo_2_O_4_ phase without other impurities. However, there is an impurity NiO phase (PDF2#96-432-0500) together with the NiCo_2_O_4_ phase, as marked in the diffraction pattern of the G2 powder. The appearance of NiO phase can be attributed to the development of a reducing atmosphere at higher fuel contents which is caused by the higher consumption of O_2_ gas. Figure 6b–g show SEM images of the G0.5, G1, and G2 samples. Although the samples synthesized by glycine fuel exhibit a porous microstructure at all fuel contents, but the particle size of NiCo_2_O_4_ and porosity characteristics are significantly dependent on the fuel contents. The average particle size increased from 18.3 to 78.6 nm for the G0.5 to G1 samples due to the higher combustion temperatures at higher fuel contents. In spite of the larger amounts of liberated gaseous product, the G2 powders have a less porous microstructure because of the premature sintering and subsequent growth of particles^22^. The cycling performance of the G0.5, G1, and G2 samples is compared in Fig. 6h. The cycling behavior of G0.5 is similar to that of G1 in which a gradual decline occurs at the start, followed by an increase at the end of 100 cycles. The final capacity of the G0.5 samples stands at 535 mAhg^−1^, which is lower than that of G1 (664 mAhg^−1^). Furthermore, the capacity fading of the G0.5 powders occurs at greater cycling numbers compared to the G1 electrode. However, the capacity of the G2 electrode continues to decrease and eventually stabilize at 130 mAhg^−1^. Unlike G0.5 and G1, the cyclic performance of the G2 electrode does not show any significant upward trend, maybe due to the inhomogeneous distribution of NiO impurity^25^. Figure 6i shows the capability rate performance of the G0.5 and G1 electrodes. The specific capacity of the G1 sample is 672, and 541 mAhg^−1^ at current density of 50, and 200 mAg^−1^, respectively, returning to 669 mAhg^−1^ when the current density is reduced back to 50 mAg^−1^. The G0.5 sample shows the same trend by demonstrating specific capacity of 526, and 431 mAhg^−1^ at current density of 50, and 200 mAg^−1^. The specific capacity of this samples increases to 570 mAhg^-1^ when the current density is returned to 50 mAg^−1^. Therefore, the G0.5 and G1 samples with the large pores and high specific surface area show a high rate capability and cycling performance. Table 1 summarizes the electrochemical performance with previously reported works. The electrochemical properties of the G1 sample are comparable with the NiCo_2_O_4_ powders synthesized by solvo/hydrothermal methods. However, the spongy G1 material is prepared by facile, simple, and efficient combustion method.

**Table 1:** Comparison of NiCo_2_O_4_ material with various morphologies.

Morphology	Final discharge specific capacity	Capacity fading (%)	Ref.
Hierarchical micro-nano hydrangea-like	863 mAhg^−1^ @ 100 mAg^−1^ for 100 cycles	47	^11^
Microellipsoids	820 mAhg^−1^ @ 100 mAg^−1^ for 100 cycles	27	^26^
Porous Nanorods	650 mAhg^−1^ @ 100 mAg^−1^ for 150 cycles	52	^27^
Hierarchical nanowire arrays	415 mAhg^−1^ @ 100 mAg^−1^ for 50 cycles	79	^28^
Bundles of long shaped particles	400 mAhg^−1^ @ 60 mAg^−1^ for 40 cycles	67	^29^
Hierarchical porous flowers	939 mAhg^−1^ @ 100 mAg^−1^ for 60 cycles	38	^30^
Spongy powders	666 mAhg^−1^ @ 100 mAg^−1^ for 100 cycles	43	G1 (This work)

**Figure 7:**
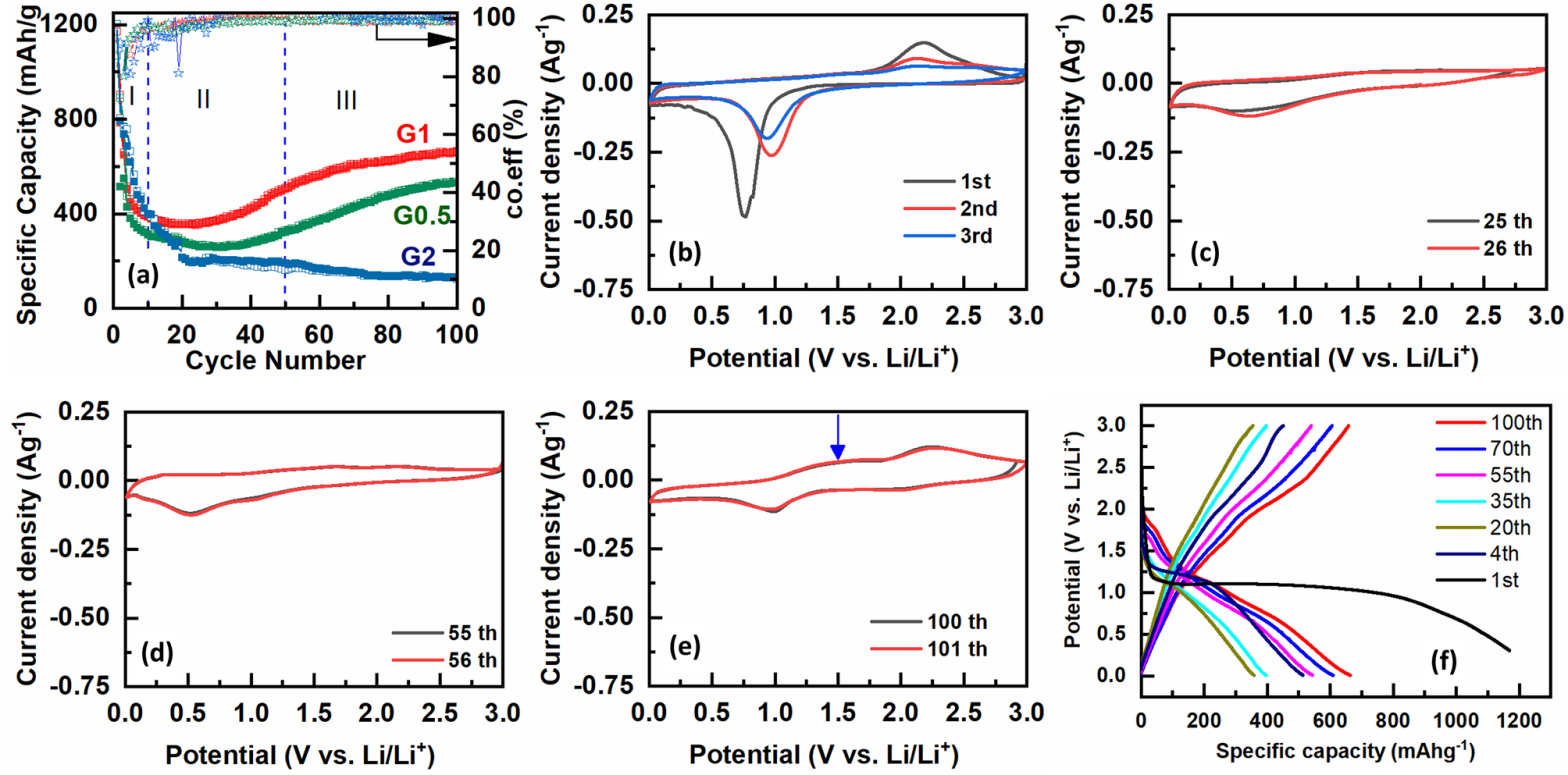
(**a**) cycling performance of and coulombic coefficient, (**b**–**e**) CV curves and (**f**) charge/discharge profiles of the G1 electrode at various cycles.

To take a closer look at cycling performance of the G1 sample, the electrochemical performance and microstructural evolution are studied at various cycling numbers. Figure 7a presents the 1st, 2nd, and 3rd CV curves in the voltage range of 0–3.0 V vs. Li/Li^+^ and at a scan rate of 0.1 mV s^−1^. According to the previous reports, the lithiation/delithiation reactions for the NiCo_2_O_4_ electrode can be presented as follows^31,32^:

1 $${\text{NiCo}}_{2}{\text{O}}_{4}+8{\text{Li}}^{+}+8{e}^{-}\to {\text{Ni}}+2{\text{Co}}+4{\text{Li}}_{2}{\text{O}}$$

2 $${\text{Ni}}+{\text{Li}}_{2}{\text{O}}\leftrightarrow {\text{NiO}}+2{{\text{Li}}}^{+}+2{e}^{-}$$

3 $${\text{Co}}+{\text{Li}}_{2}{\text{O}}\leftrightarrow {\text{CoO}}+2{\text{Li}}^{+}+2{e}^{-}$$

4 $${\text{CoO}}+\frac{1}{3}{\text{Li}}_{2}{\text{O}}\leftrightarrow \frac{1}{3}{\text{Co}}_{3}{\text{O}}_{4}+\frac{2}{3}{\text{Li}}^{+}+\frac{2}{3}{e}^{-}$$

The strong cathodic peak at about 0.75 V vs. Li/Li^+^ in the 1st scan can be attributed to the formation of the SEI compounds and metallic Co and Ni at the electroactive material’s surface [Eq. (1)]. Because of the irreversible reactions that took place at the initial cycle, the reduction peak is shifted and weakened to a higher potential (1 V vs. Li/Li^+^) at 2nd and 3rd scans^30,33^. The oxidation peak at about 2.2 V vs. Li/Li^+^ upon lithium extraction process is related to the oxidation peak of Co^0^ to Co^3+^. The oxidation peaks are broadened and weakened by continuous cycling^34^. The cyclic voltammetry curves don't overlap very well from the second cycle onwards because of the poor reversibility of the electrochemical reactions during the initial charging and discharging cycles. For 25 and 26th cycle, the anodic peaks are broadened, weakened, and hardly detectable in Fig. 7b. However, there is a specific cathodic peak which is slightly intensified by cycling. The anodic and cathodic peaks appear together with the increase of specific capacity as the cycling number reaches to 55th (Fig. 7c). The anodic peak at 1.5 V vs. Li/Li^+^ is related to the oxidation of Ni^0^ to Ni^2+^ during lithium extraction process^35^. Furthermore, the redox peaks are intensified upon reaching the 100th cycle (Fig. 7d). The cyclic voltammetry curves approximately overlap by cycling, showing the improvement of reversibility redox reactions.

As previously reported in the literature^36^, upon the first lithiation/delithiation process, the Li ions are inserted into and extracted from the NiCo_2_O_4_ material structure, leading to the regional structural collapses by the conversion reaction. The deep lithiation results in the agglomerations and heterogeneous reactions, especially in the large, polycrystalline, porous NiCo_2_O_4_ particles. The polarization and the structural changes of electroactive materials are responsible for the dramatic capacity loss according to the progressive broadening and severe decay of redox peaks. In other words, the fading of electrochemical reversibility can be attributed to the irreversible formation of some metallic Co and Ni species that continues to accrete the electrode during subsequent cycling^35^. However, the appearance of Ni^0^/Ni^2+^ redox peaks is associated with significantly reduced particle size, as will be shown by SEM, immensely improving the lithium-ion diffusion kinetics by providing more sites for lithium-ion storage via increasing the surface area^37^.

The galvanostatic charge/discharge profiles of the G1 electrode are also shown for their 1st, 2nd, 3rd, 15th, and 100th cycles in Fig. 7. The initial discharge capacity of 1198 mAhg^−1^ decreased up to 508 mAhg^−1^ which can be attributed to the irreversible formation of the SEI and metallic Co and Ni layers. The initial discharge voltage plateau at 1.1 V is related to the conversion mechanism of NiCo_2_O_4_, including SEI and metallic Co and Ni formation^35^. The plateau profile changes to a steeper slope after the 35th discharge cycle which the discharge capacity is reduced to 404 mAhg^−1^, owing to the structural and textural modifications during the progressive electrochemical cycling^38^. The plateau profile has transformed into a long potential slope for both the charge and discharge plateau upon the 100th cycle which is related to the increase of discharge and charge capacity up to 658 and 664 mAhg^-1^, respectively.

**Figure 8:**
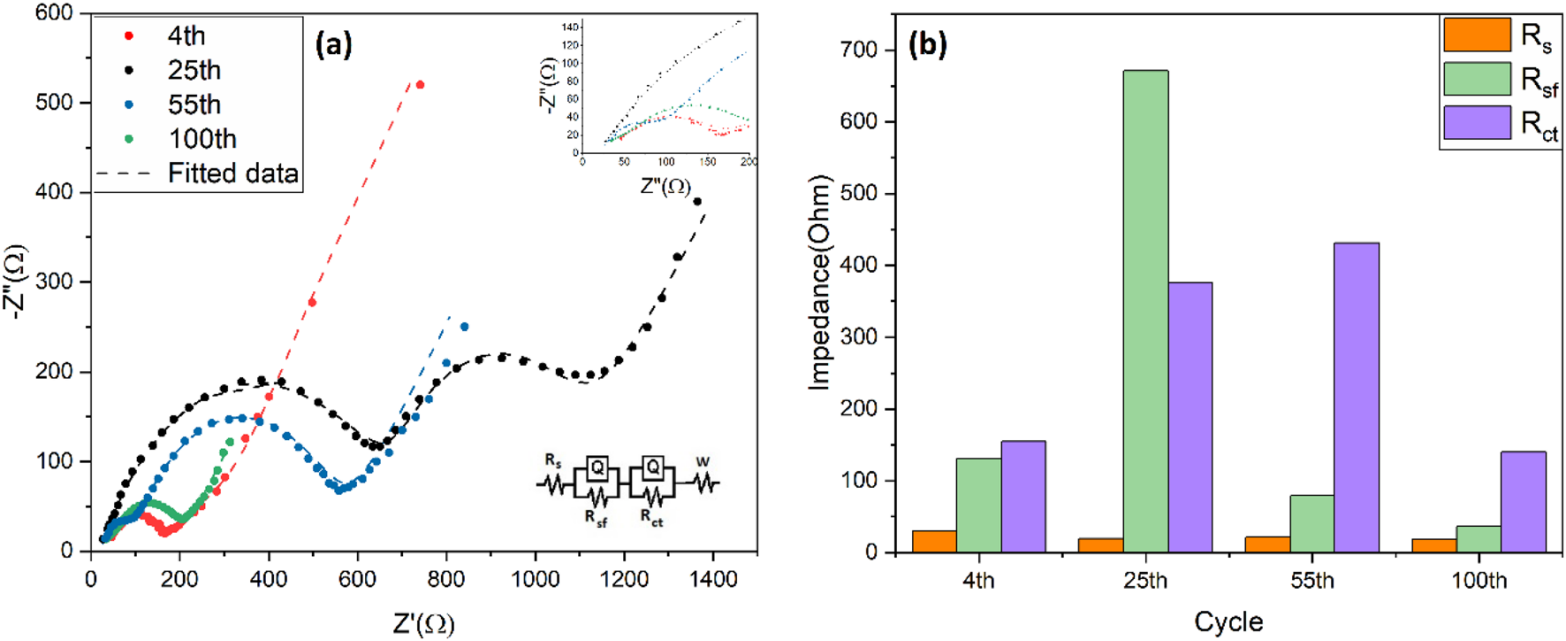
(**a**) Nyquist plots and the fitted values of the G1 electrode after different cycles, and (**b**) The values of Rs, Rsf, and Rct vs. cycle number.

**Figure 9:**
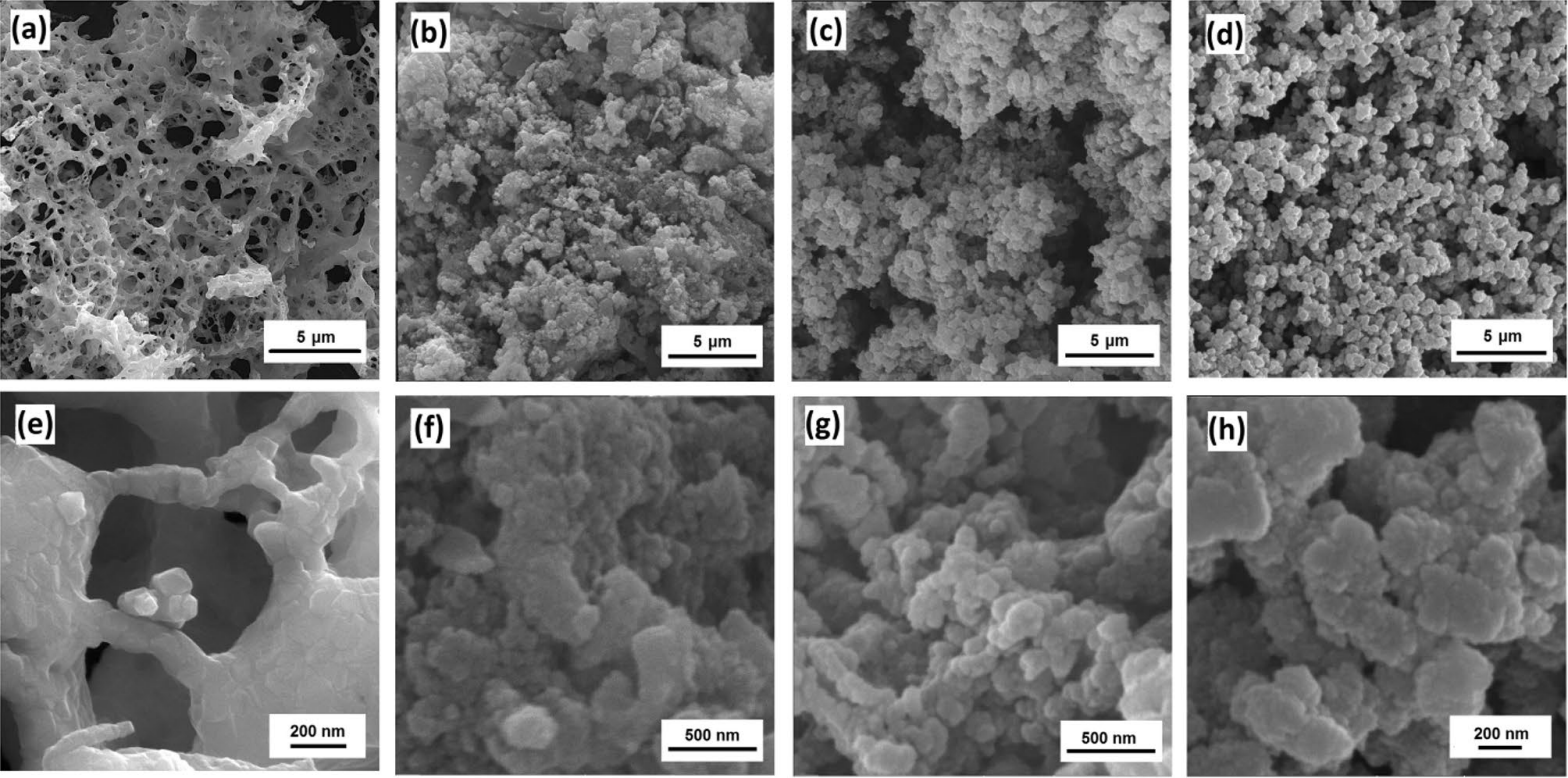
SEM images of the G1 electrode before cycling (**a**, **e**) and after 4th (**b**, **f**), 25th (**c**, **g**), and 100th cycle (**d**, **h**).

The electrochemical impedance spectra (EIS) of the G1 powders after 4, 25, 55, and 100th cycles are given in Fig. 8a to study the interfacial charge-transfer and Li^+^ diffusion processes. The Nyquist plots (− Zʺ vs. Z′) include two partially overlapped semicircles and an inclined straight line in the high, medium, and low frequency regions, respectively^39^. The ohmic resistance of the electrolyte (R_s_) is represented by Z_0_ intercept at the high-frequency region. The semicircle in the high-frequency range is associated with the solid-electrolyte interface resistance (R_sf_), while the interface charge transfer resistance (R_ct_) can be calculated according to the medium-frequency semicircle. The Warburg impedance as inclined line in the low-frequency region is related to the solid state diffusion of Li^+^ ions into the electroactive structure^39^. The values of R_s_, R_sf_, R_ct_ versus cycle number are summarized in Fig. 8b. At initial cycles, the R_sf_ (133 Ω) and R_ct_ (150 Ω) are relatively small. With cycling, up to the minimum specific capacity (25th cycle), the R_sf_ and R_ct_ increase to 673 Ω and 325 Ω, respectively. Interestingly, the value of R_sf_ is reduced to 71 Ω at 55th cycle as the specific capacity is recovered. The occurrence of these two opposite trends can be attributed to the microstructural evolution of the electrode material^40^. Figure 9 shows the highly porous structure of G1 powders (Fig. 9a,e) was destroyed during the first four cycles, giving way to an agglomerated microstructure (Fig. 9b,f). By cycling, the particles become smaller with a high surface-to-volume ratio, leading to the higher formation of SEI layer^41^ which is well consistent with the large R_sf_ at 25th cycle. However, the clusters of nanoparticles are joined together to form coarse clusters at the higher cycle numbers of 55th (Fig. 9c,g) and 100th (Fig. 9d,h), resulting in the lower surface-to-volume ratio and then lower R_sf_ value.

Finally, Stage I (Fig. 7a) is mainly distinguished by the strong drop of specific capacity caused by the initial reactions with the electrolyte and build-up of the SEI and metallic layer^35,38^. Simultaneously, the phase transitions during electrochemical oxidization of Co^0^ and Ni^0^ to CoO and NiO result in the pulverization of NiCo_2_O_4_ particles with initial contact losses between NiCo_2_O_4_ particles and black carbon as conductive agent. For stage II (Fig. 7a), the columbic efficiency increases and stabilize up to 100% which is mainly attributed to slow and continuous penetration of the electrolyte through the inner parts of the porous network, gradually penetrating the electrode as well as re-establishment of the contact between pulverized NiCo_2_O_4_ particles (Fig. 9c,g). The three-dimensional network of porosity between NiCo_2_O_4_ particles enhance the cycling stability by accommodating volume expansion stresses^42^. The gradual enhancement of capacity in stage III can be ascribed to the consecutive formation and decomposition of a gel-like film containing Li_2_O- and some other lithium compounds, facilitating the transportation of lithium ions. It was reported that the metallic particles are dispersed within the gel-like film, thereby preventing their agglomeration^43,44^.

## Conclusion

The single phase NiCo_2_O_4_ powders were successfully synthesized by solution combustion method using l-lysine, glycine, and urea as organic fuel. The NiCo_2_O_4_ powders prepared by glycine and l-lysine fuel showed the porous microstructure owing to the fast liberation of large amounts of gas products. However, the urea fuel led to the agglomerated NiCo_2_O_4_ nanoparticles on account of the intermediate precipitation of hydroxides during gelation process. A capacity recovery behavior was observed in the porous NiCo_2_O_4_ powders, while the behavior was previously reported for the transition metal oxide–carbon composite powders. The capacity recovery behavior of the porous NiCo_2_O_4_ powders was rationalized by various electrochemical tests.
